# MRI Findings Suggestive of Trigeminal and Vestibulocochlear Nerve Involvement in Miller Fisher Syndrome

**DOI:** 10.3390/reports9040325

**Published:** 2026-09-25

**Authors:** Ayuhei Nako, Koji Hayashi, Mamiko Sato, Hiroaki Maeda, Yasutaka Kobayashi

**Affiliations:** 1 Department of Rehabilitation Medicine, Fukui General Hospital, Fukui 910-8561, Japansatomoko@f-gh.jp (M.S.);; 2 Graduate School of Health Science, Fukui Health Science University, Fukui 910-3190, Japan

**Keywords:** Miller Fisher syndrome, trigeminal nerve, vestibulocochlear nerve, magnetic resonance imaging, ophthalmoplegia

## Abstract

Miller Fisher syndrome (MFS) is a variant of Guillain–Barré syndrome characterized by a triad of ophthalmoplegia, ataxia, and areflexia, typically associated with anti-GQ1b antibodies. While cranial nerve enhancement is sometimes seen on high-resolution MRI, involvement of the trigeminal (CN V) and vestibulocochlear (CN VIII) nerves is rare. We report a 62-year-old woman presenting with diplopia, preceded by ophthalmalgia, nasal congestion, and vertigo. Neurological assessment revealed right-sided ptosis, total ophthalmoplegia, sluggish pupillary reflexes, diplopia without nystagmus, bilateral limb ataxia, hyporeflexia, and impaired vibration and joint-position sensation. Serology was positive for anti-GQ1b antibodies. Contrast-enhanced MRI demonstrated signal changes suggestive of CN V and CN VIII involvement. Although cerebrospinal fluid showed no albuminocytologic dissociation, she was diagnosed with MFS. She received immunotherapy, including intravenous immunoglobulin therapy. While her vertigo, ophthalmalgia and ataxia significantly improved, ophthalmoplegia persisted. The patient was discharged on hospitalization day 44 with residual ocular motility deficits. In this patient, orbital pain with cranial autonomic features (ocular pain, nasal congestion) and vertigo of uncertain localization acted as misleading early signs. This case illustrates an unusual MFS presentation where prodromal CN V and VIII symptoms preceded classic features, highlighting the need for careful clinical evaluation when sensory and vestibular symptoms precede classic features.

A 62-year-old woman presented with sudden-onset diplopia and progressive gait unsteadiness, designated as hospitalization Day 0. Five days prior to admission (Day-5), she developed upper respiratory symptoms that resolved with over-the-counter medications. Two days prior to admission (Day-2), she experienced severe retro-orbital pain radiating to the neck, accompanied by nasal congestion and conjunctival injection. Rotational vertigo accompanied by nausea appeared one day prior (Day-1) and intensified on admission, accompanied by total external ophthalmoplegia and gait instability. Vital signs were unremarkable, except for elevated blood pressure (167/108 mmHg). Physical assessment revealed retro-orbital pain and head heaviness. Neurological assessment revealed right ptosis, total external ophthalmoplegia, diplopia without nystagmus, and sluggish pupillary light reflexes. Muscle strength was preserved without involuntary movements, but deep tendon reflexes were globally diminished. Coordination testing demonstrated prominent limb ataxia, postural instability, and gait unsteadiness. Sensory examination showed diminished joint position and vibration senses in the toes. Formal audiologic, vestibular, or quantitative trigeminal sensory threshold examinations were not performed upon initial presentation. Blood tests were unremarkable, including leukocyte count and C-reactive protein, except for mildly elevated aspartate aminotransferase (37 U/L; reference range: 13–30 U/L) and gamma-glutamyl transferase (188 U/L; reference range: 9–32 U/L) levels. Subsequent serological testing was positive for anti-GQ1b IgG antibody and negative for anti-GM1 antibody. On hospitalization Day 0, cerebrospinal fluid (CSF) analysis showed a normal cell count (1/µL; reference range: 0–5/µL) and protein level (24.8 mg/dL; reference range: 15–45 mg/dL), showing no albuminocytological dissociation. Nerve conduction studies were normal. Contrast-enhanced brain MRI on Day 0 revealed signal changes suggestive of involvement of the trigeminal (CN V) and vestibulocochlear (CN VIII) nerves. Diagnosed with anti-GQ1b-positive Miller Fisher syndrome, the patient received IVI starting on Day 0, followed by intravenous methylprednisolone pulse therapy. Retro-orbital pain, vertigo, and ataxia resolved within 3 days after initiating IVIg, but ophthalmoplegia persisted. She was discharged on the 44th hospital day with residual ocular motility deficits.

**Figure 1:**
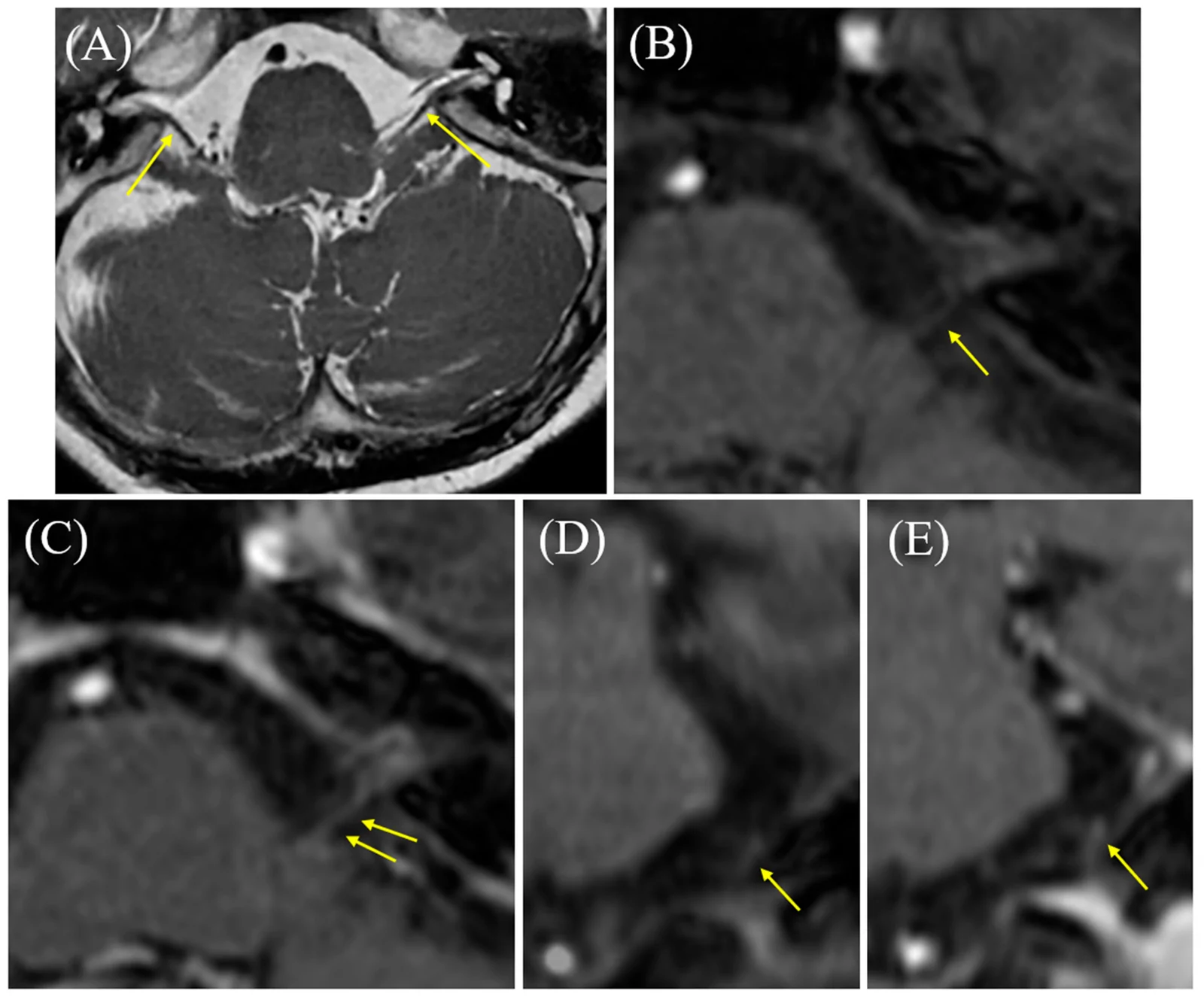
Brain magnetic resonance imaging (MRI) at the level of the vestibulocochlear nerves. Images were acquired using a 1.5-T MRI scanner (SIGNA™ Artist Evo; GE Healthcare) before/after intravenous administration of gadobutrol (5 mL [3.0236 g]) with a 3-min post-contrast delay. (**A**) Axial 3D T2-weighted fast spin-echo image with variable refocusing flip angles (T2-CUBE; acquired voxel size: 0.6 × 0.6 × 0.8 mm^3^, reconstructed slice thickness: 0.2 mm); (**B**) axial 3D time-of-flight spoiled gradient-recalled echo image (3D TOF-SPGR; acquired voxel size: 0.6 × 0.6 × 0.3 mm^3^, reconstructed slice thickness: 0.2 mm) serving as a pre-contrast anatomical reference; (**C**) axial contrast-enhanced 3D T1-weighted image with variable refocusing flip angles (acquired voxel size: 0.6 × 0.3 × 0.8 mm^3^, reconstructed slice thickness: 0.2 mm); (**D**) coronal 3D TOF-SPGR; and (**E**) coronal contrast-enhanced T1-weighted image. Panels (**B**–**E**) focus on the left vestibulocochlear nerve to evaluate the degree of enhancement. Yellow arrows indicate the cisternal segments of the vestibulocochlear nerves bilaterally. Signal intensity changes suggestive of subtle contrast enhancement are observed along the cisternal segment of the left CN VIII on the post-contrast images (**C**,**E**) compared with the pre-contrast references (**B**,**D**), although potential overlap with adjacent facial nerve (CN VII) fibers or microvascular structures must be considered. The clinical spectrum of anti-GQ1b antibody syndrome has evolved into a recognized continuum of immune-mediated neuropathies, far exceeding the classic MFS triad. This spectrum encompasses diverse phenotypes, ranging from Bickerstaff brainstem encephalitis (BBE) at the central nervous system pole to regional or incomplete variants such as acute isolated ophthalmoparesis (AO), acute ataxic neuropathy (AAN), and the pharyngeal–cervical–brachial (PCB) variant [1,2,3,4]. In the present case, the patient experienced prominent vertigo prior to the emergence of the classic MFS triad (extraocular muscle paralysis, ataxia and hyporeflexia). Initially, attributing her symptoms to a benign, self-limiting condition, she opted for conservative observation. However, her initial presentation—characterized by sudden-onset rotational vertigo, nausea, and postural instability—suggests a possible association with the left CN V/VIII signal findings [1,2,3]. Identifying this subtype is clinically critical because AVS presentations frequently deviate from traditional neurological expectations. For instance, while deep tendon areflexia is a hallmark of classic MFS, studies show that 73% of patients with anti-GQ1b-associated AVS maintain normal deep tendon reflexes, and 100% of tested patients (4/4) exhibit normal results in standard nerve conduction studies (NCS) [2,3]. As demonstrated in this case, vertigo occurring before the onset of the classic triad can easily mimic benign vestibular disorders, potentially warranting careful diagnostic consideration.

**Figure 2:**
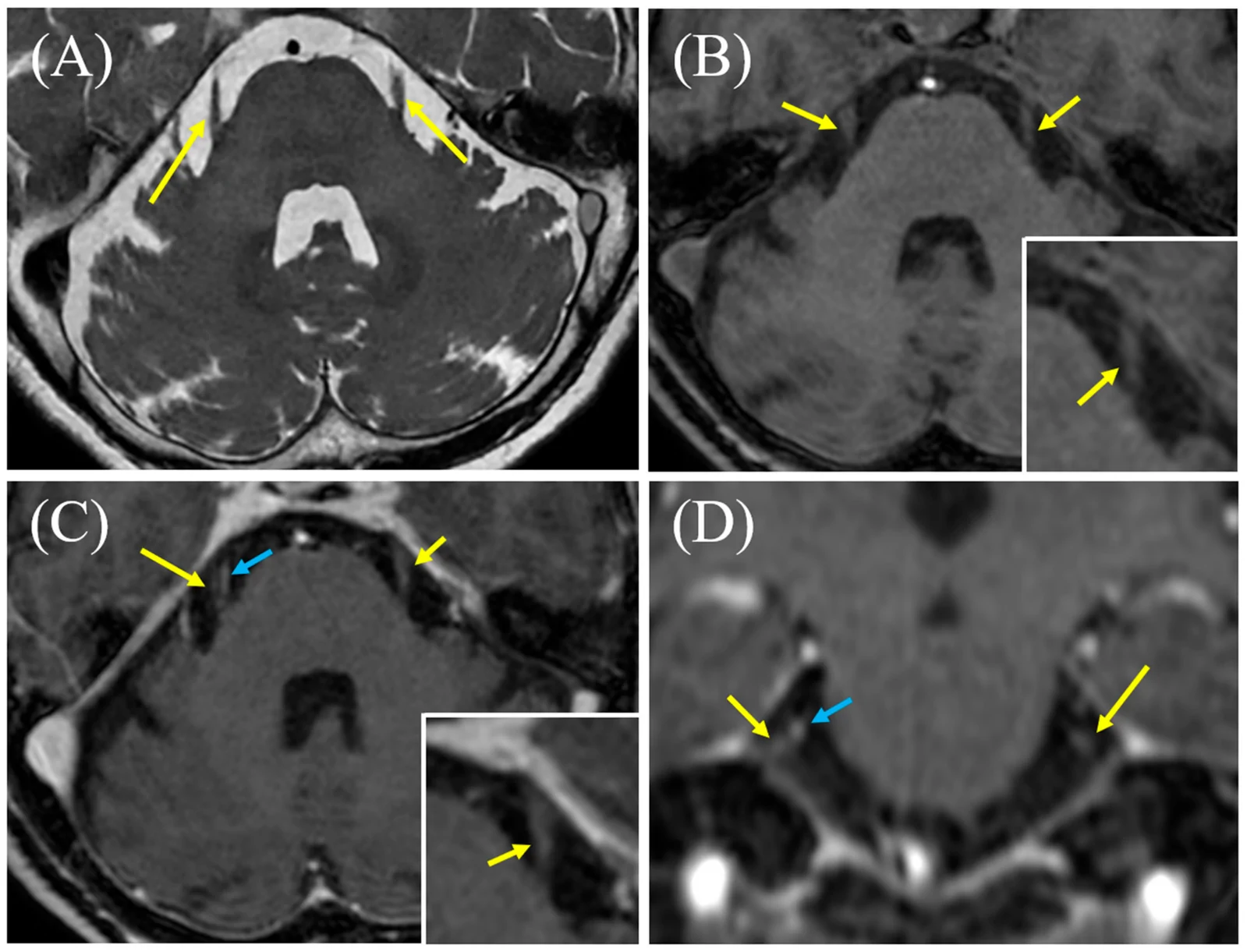
Brain MRI at the level of the trigeminal nerves. Images were acquired on a 1.5-T MRI scanner (SIGNA™ Artist Evo; GE Healthcare) before/after intravenous administration of gadobutrol (5 mL [3.0236 g]). (**A**) Axial 3D T2-weighted fast spin-echo image with variable refocusing flip angles (T2-CUBE; reconstructed slice thickness: 0.2 mm); (**B**) axial 3D time-of-flight spoiled gradient-recalled echo image (3D TOF-SPGR; acquired voxel size: 0.6 × 0.6 × 0.3 mm^3^, reconstructed slice thickness: 0.2 mm) used as a pre-contrast anatomical reference; and (**C**) axial and (**D**) coronal contrast-enhanced T1-weighted images with variable refocusing flip angles (reconstructed slice thickness: 0.2 mm and 2.0 mm, respectively). Yellow arrows highlight the cisternal segment of the left trigeminal nerve, where signal alteration suggestive of contrast enhancement is noted on post-contrast images (**C**,**D**) compared with the pre-contrast state (**B**). Blue arrows in (**C**) and (**D**) indicate an adjacent superior cerebellar artery branch running close to the right trigeminal nerve. The left trigeminal nerve was confirmed to be anatomically distinct from nearby vessels, minimizing partial-volume artifacts. Insets in (**B**) and (**C**) present magnified views of the cisternal segment of the left CN V. While cranial nerve enhancement in MFS most frequently involves the oculomotor and abducens nerves, trigeminal nerve (CN V) involvement remains a less characterized finding [4]. A recent high-resolution MRI study by Lee et al. documented trigeminal enhancement in a patient presenting with facial hypoesthesia [4], suggesting a potential breakdown of the blood–nerve barrier mediated by anti-GQ1b antibodies [4,5]. However, reports providing a detailed temporal and clinical correlation between specific trigeminal nociceptive/autonomic symptoms and radiographic evidence remain scarce. Indeed, while clinical trigeminal dysfunction has been documented in cases such as that by Deng et al., these reports often lack corresponding radiographic evidence of CN V involvement [6]. Our patient exhibited prodromal symptoms suggestive of trigeminal involvement prior to the onset of classic ophthalmoplegia—specifically, severe ophthalmalgia and nasal congestion. The ophthalmalgia was particularly striking, manifesting as paroxysmal, severe pain in the bilateral orbital and supraorbital regions, accompanied by cranial autonomic signs including nasal congestion and conjunctival injection, without overt facial sensory deficits. While Ejma et al. previously described clinical trigeminal hyperaesthesia and burning sensations without corresponding imaging findings [7], the present case illustrates a possible clinical–radiographic association using contrast-enhanced MRI. The paroxysmal orbital pain and autonomic signs observed in our patient lend clinical support to the hypothesis proposed by Friedman and Potts, which suggests that anti-GQ1b antibodies may directly affect the trigemino-vascular pain pathway [7,8]. Furthermore, this case highlights an important diagnostic pitfall; because MFS typically develops 1 to 3 weeks after the antecedent infection including an upper respiratory tract infection, early CN V symptoms such as nasal congestion and periorbital discomfort can easily be misinterpreted as lingering signs of a common cold or viral rhinosinusitis. This clinical mimicry may lead to an underestimation of the true incidence of trigeminal involvement. By capturing signal alteration suggestive of left CN V involvement alongside these specific clinical features, this case provides an instructive example that broadens our understanding of the multi-cranial nerve spectrum in anti-GQ1b antibody syndrome.

**Figure 3:**
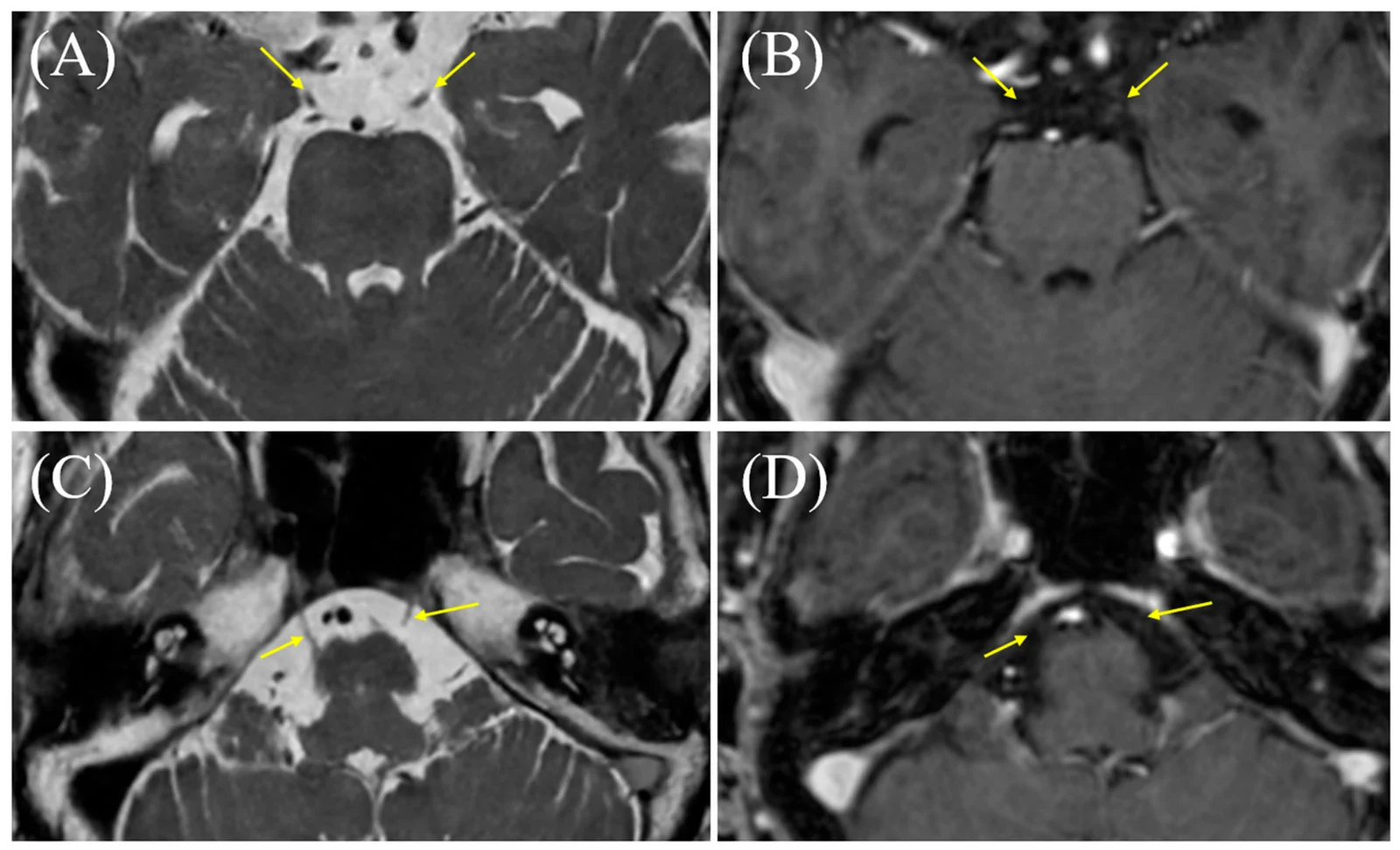
Brain magnetic resonance imaging (MRI) at the levels of the oculomotor and abducens nerves. Images were acquired on a 1.5-T MRI scanner (SIGNA™ Artist Evo; GE Healthcare). Axial 3D T2-weighted fast spin-echo images (T2-CUBE; reconstructed slice thickness: 0.2 mm) (**A**,**C**) and contrast-enhanced 3D T1-weighted images (3 min post-gadobutrol, reconstructed slice thickness: 0.2 mm) (**B**,**D**) at the levels of the oculomotor (CN III; (**A**,**B**)) and abducens (CN VI; (**C**,**D**)) nerves. Yellow arrows indicate the respective nerve pathways. Despite the patient’s total ophthalmoplegia, no conspicuous abnormal enhancement was detected along the oculomotor or abducens nerves on this examination; such clinical–radiographic dissociation remains one possible interpretation.

## Data Availability

The data presented in this study are available on request from the corresponding author. Due to patient privacy and ethical considerations, the data are not publicly accessible.
